# Evolution of Cytochrome P450 Enzymes and Their Redox Partners in Archaea

**DOI:** 10.3390/ijms24044161

**Published:** 2023-02-19

**Authors:** Phelelani Erick Ngcobo, Bridget Valeria Zinhle Nkosi, Wanping Chen, David R. Nelson, Khajamohiddin Syed

**Affiliations:** 1 Department of Biochemistry and Microbiology, Faculty of Science and Agriculture, University of Zululand, KwaDlangezwa 3886, South Africa; 2 Department of Molecular Microbiology and Genetics, University of Göttingen, 37077 Göttingen, Germany; 3 Department of Microbiology, Immunology and Biochemistry, University of Tennessee Health Science Center, Memphis, TN 38163, USA

**Keywords:** evolution, cytochrome P450 monooxygenases, ferredoxins, bacteria, eukarya, lateral gene transfer, operon, plasmids, phylogenetic analysis

## Abstract

Cytochrome P450 monooxygenases (CYPs/P450s) and their redox partners, ferredoxins, are ubiquitous in organisms. P450s have been studied in biology for over six decades owing to their distinct catalytic activities, including their role in drug metabolism. Ferredoxins are ancient proteins involved in oxidation-reduction reactions, such as transferring electrons to P450s. The evolution and diversification of P450s in various organisms have received little attention and no information is available for archaea. This study is aimed at addressing this research gap. Genome-wide analysis revealed 1204 P450s belonging to 34 P450 families and 112 P450 subfamilies, where some families and subfamilies are expanded in archaea. We also identified 353 ferredoxins belonging to the four types 2Fe-2S, 3Fe-4S, 7Fe-4S and 2[4Fe-4S] in 40 archaeal species. We found that bacteria and archaea shared the CYP109, CYP147 and CYP197 families, as well as several ferredoxin subtypes, and that these genes are co-present on archaeal plasmids and chromosomes, implying the plasmid-mediated lateral transfer of these genes from bacteria to archaea. The absence of ferredoxins and ferredoxin reductases in the P450 operons suggests that the lateral transfer of these genes is independent. We present different scenarios for the evolution and diversification of P450s and ferredoxins in archaea. Based on the phylogenetic analysis and high affinity to diverged P450s, we propose that archaeal P450s could have diverged from CYP109, CYP147 and CYP197. Based on this study’s results, we propose that all archaeal P450s are bacterial in origin and that the original archaea had no P450s.

## 1. Introduction

Cytochrome P450 monooxygenases (CYPs/P450s) are a superfamily of heme-containing enzymes discovered nearly six decades ago [1,2,3,4,5]. In the name, “cytochrome” indicates a heme-bound protein, “450” refers to the feature that these reduced proteins with carbon-monoxide bound absorb light at 450 nm wavelength and “monooxygenases” indicates their enzymatic reaction, i.e., incorporation of one oxygen atom into the substrates [1,2,3,4,5]. Although these enzymes are named monooxygenases, research has shown they perform diverse enzymatic reactions with regio- and stereo-selectivity [6,7,8]. These unique catalytic properties led to the exploration of the applications of these enzymes in all areas of biology [9,10,11,12,13].

A unique nomenclature and classification system has been developed for P450s [14,15,16,17]. The nomenclature system begins with the prefix “CYP” for cytochrome P450 monooxygenase, followed by an Arabic numeral designating the family, a capital letter representing the subfamily and an Arabic digit specifying the individual P450 in a family. The annotation/classification criteria include assigning family and subfamily with >40% identity belonging to the same family and all P450s with >55% identity belonging to the same subfamily [14,15,16,17].

P450s play an important role in the primary and secondary metabolism of organisms; thus, they can be found in archaea, bacteria and eukarya (Table 1) [18,19]. Unexpectedly, P450s were found in non-living entities such as viruses (Table 1) [20,21]. The evolution of P450s is linked to sterol biosynthesis, where the CYP51-catalyzed demethylation reaction is widely accepted as an ancestral reaction and thus CYP51 is a possible ancestor of all known eukaryotic P450s [9]. A recent study has shown that CYP51 evolved in bacteria and then moved into eukarya [22]. The evolution of viral P450s has also been investigated, where the apparent donors of the P450s are unclear [21]. Information on the evolution of P450s in bacteria, eukarya and viruses is available, but not in archaea. Archaea have P450s and one of the P450s, CYP119 from *Sulfolobus solfataricus*, has been subjected to intense investigation due to its thermophilic properties [23,24,25,26,27,28].

All P450s, except for self-sufficient P450s, need electrons for their enzymatic action, which are transferred by redox proteins [29]. Studies indicated the presence of functional ferredoxins and their reductase, known as 2-oxoacid: ferredoxin oxidoreductase (OFOR) in archaea [30]. The OFOR consists of alpha and beta subunits and the genes encoding these subunits often reside in proximity [31]. Ferredoxins are iron-sulfur (Fe-S) cluster proteins that evolved during early chemical evolution [32,33]. These proteins are involved in the transfer of electrons in oxidation-reduction reactions, including P450 reactions [29]. Ferredoxins, like other Fe-S cluster proteins, are classified into different types based on the number of Fe-atoms in their cluster, such as 2Fe-2S, 3Fe-4S, 4Fe-4S, 7Fe-8S (3Fe-4S and 4Fe-4S) and 2[4Fe-4S]) [29]. Ferredoxins are further classified into subtypes based on the characteristic spacing between the cysteine amino acids of the Fe-S binding motif [34]. A recent study provided preliminary information on ferredoxins in archaea [34]. The research showed that several archaeal ferredoxin subtypes are also present in bacteria and eukarya [34].

Considering there is no information concerning the evolution of archaeal P450s and their redox partners, this study is aimed at addressing this research gap. In this study, we performed genome-wide data mining, annotation and phylogenetic analysis of P450s and their redox partners in archaea. Also, we provided evidence on the evolution of archaeal P450s from bacteria, possibly by lateral gene transfer via plasmids.

## 2. Results and Discussion

### 2.1. Archaea Has the Lowest P450 Diversity

**Figure 1:**
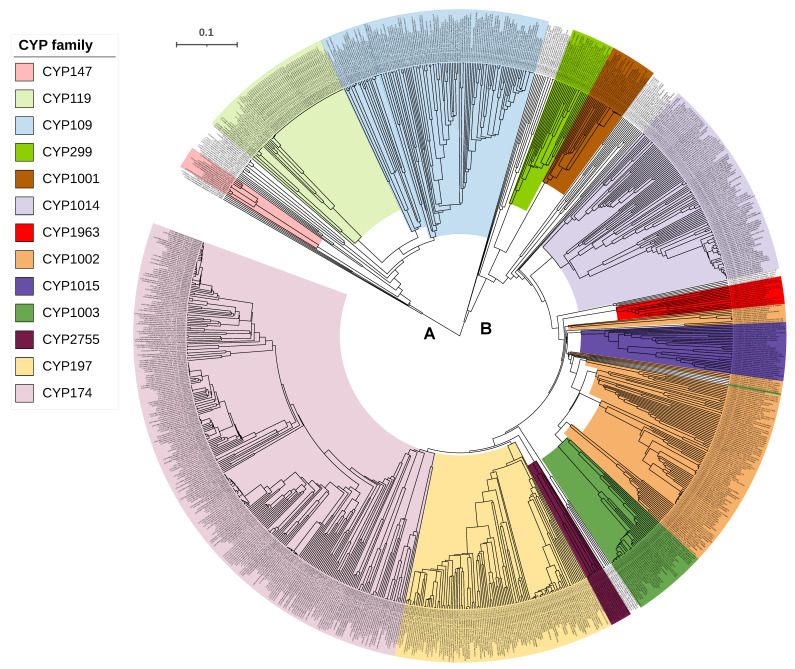
Phylogenetic analysis of archaeal P450s. Major P450 families and the P450 families that are common between bacteria and archaea are displayed in different colors. The tree branch length is indicated as the scale bar in the upper left corner. A and B represent the two main branches of the tree. The tree is constructed with P450 sequences presented in Table S1 by the alignment of MAFFT v6.864 [36] and inferred by the Trex web server [37].

**Figure 2:**
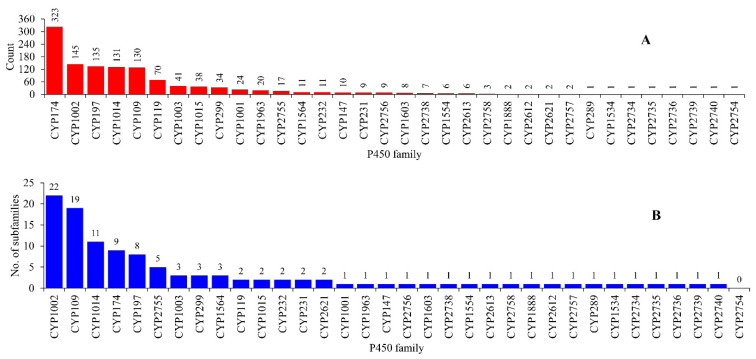
Comparative analysis of P450 families (**A**) and subfamilies (**B**) in archaea. The numbers next to the bars represent the member count for that family or subfamily. Detailed information is provided in Table S2.

Genome data mining and annotation of P450s in archaeal sequences available at Joint Genome Institute Integrated Microbial Genomes and Microbiomes (JGI IMG/M) [35] revealed the presence of 1209 P450s, including five short protein sequences without P450 motifs (known as P450 fragments) (Figure 1 and Table S1). All 1204 P450s, except for five P450 fragments, can be grouped into 34 P450 families and 112 P450 subfamilies (Figure 2 and Table S2). The number of P450 families found in archaea is the lowest compared to bacteria and eukarya (Table 1) [18], indicating that archaea have the lowest P450 diversity. A P450 family saturation analysis revealed that P450 families are nearing saturation in archaea (Figure S1). The lowest P450 diversity and P450 family saturation in archaea are somewhat surprising, as this was not the case for bacteria and eukarya [18].

### 2.2. Some P450 Families and Subfamilies Are Expanded in Archaea

Among the P450 families found in archaea, CYP174 had the highest number of members (323 P450s), followed by CYP1002 (145 P450s), CYP197 (135 P450s), CYP1014 (131 P450s) and CYP109 (130 P450s) (Figure 2 and Table S2). A total of 19 P450 families have less than 10 members and 10 families have between 10–70 members (Figure 2 and Table S2). This suggests that CYP174, CYP1002, CYP197, CYP1014 and CYP109 are expanded (the presence of the same P450 in many species) in archaea and, thus, possibly play an important role in these species. This phenomenon is also observed at the subfamily level, where members belonging to a particular subfamily are more highly populated in archaea (Figure 2 and Table S2). Among P450 families, CYP1002 has the highest number of subfamilies (22), followed by CYP109 (19), CYP1014 (11) and CYP174 (9) (Figure 2 and Table S2). Despite having the highest number of members, CYP174 had only nine subfamilies, with subfamilies CYP174B and CYP174A expanded with 182 and 96 members (Table S2). Subfamily A is expanded with 60 members in CYP119. This suggests that these subfamilies possibly play an important role and thus are expanded in archaea. Overall, the subfamily level diversity observed for archaea is lowest compared to bacteria and eukarya [18].

### 2.3. Plasmid-Mediated Lateral Transfer of P450s from Bacteria to Archaea

P450 comparisons revealed three P450 families are shared by archaea and bacteria but no P450 family is shared by archaea and eukarya. The commonly shared archaeal and bacterial P450 families are CYP109, CYP147 and CYP197. The copresence of these P450 families was observed in both the archaea and bacteria (Table S3). CYP109 and CYP197 are found in the same species 35 times, CYP109 and CYP147 are found in the same species two times and CYP197 and CYP147 are located in the same species three times (Table S3). Furthermore, the genus *Myxococcus* has all three families but not all three in the same species (Table S3). The copresence of these P450 families in both archaea and bacteria indicates the possibility that these P450 families came from bacteria to archaea by lateral gene transfer.

**Figure 3:**
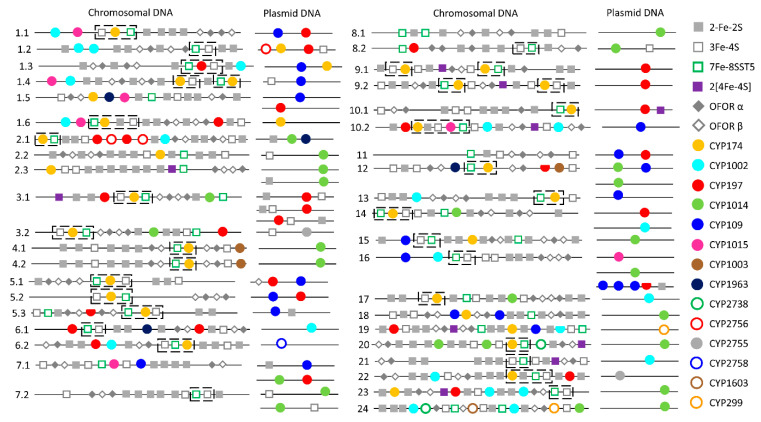
Schematic representation of P450s, ferredoxins and ferredoxin reductase (OFOR) on chromosomal and plasmid DNA from 40 archaeal species. The numbers 1 to 24 represent archaeal species belonging to different genera and subsection numbers such as 1.1, 2.1, etc., represent species in a genus. P450s are presented with circles (filled or empty), where a half-filled circle indicates a P450 fragment. Ferredoxins are represented per their iron-sulfur cluster type and OFOR with alpha and beta subunits. A dashed line box shows the association between CYP174, 3Fe-4SST1 and 7Fe-8SST5. P450s, ferredoxins and ferredoxin reductase alpha and beta subunits. Detailed information is presented in Tables S5 and S6.

It is well-known that plasmids play a role in carrying genes from one organism to another and archaeal plasmids have been known to shuttle genes from bacteria and eukarya [38]. To find out if any P450s are on archaeal plasmids, we analyzed archaeal plasmids for P450s (Figure 3 and Table S4). In total, 63 P450s were found on plasmids belonging to 40 archaeal species (Figure 3 and Tables S4 and S5). The *Halocatena* sp. AD-1 plasmid (unnamed3: NZ_CP096022.1/CP096022.1) had the highest number of members (four), followed by three members on *Halorussus halophilus* ZS-3 plasmid (unnamed1: NZ_CP044524.1/CP044524.1) (Figure 3 and Table S5). A total of 11 P450 families (out of 34 P450 families in archaea) are found on the archaeal plasmids (Figure 3 and Table S5). Among archaeal plasmid P450 families, CYP1014 had the most members (19), followed by CYP197 and CYP109 (15 members each), CYP1002 (four members), CYP174 (3 members), CYP2755 (2 members) and P450 families CYP299A18, CYP2758A1, CYP2756A5, CYP1963A15 and CYP1015B2 had a single member (Figure 3 and Table S5).

**Figure 4:**
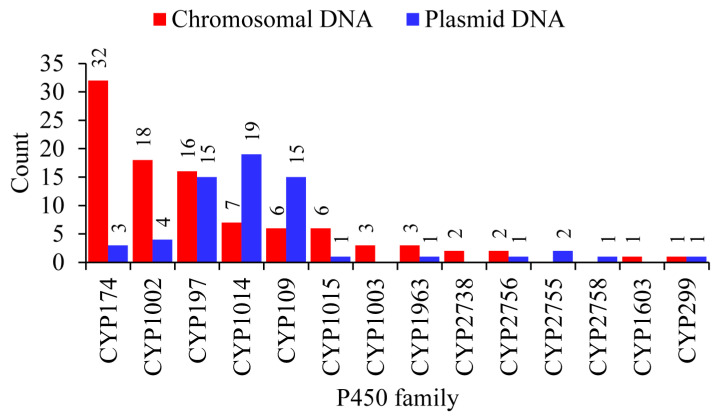
Comparative analysis of P450 families between chromosomal and plasmid DNA from 40 archaeal species. The numbers next to the bars represent the member count for that family. Detailed information is provided in Tables S5 and S6.

P450s of the same family were found on both plasmids and chromosomes and in species belonging to the same genus; some plasmids have a P450 and the same P450 is absent on plasmids but present on the chromosome in some species, indicating the transfer of P450s between plasmids and chromosomal DNA (Figure 3 and Tables S5 and S6). Comparative analysis of P450 families revealed a pattern where certain P450 families are expanded after being transferred from plasmids to chromosomes in archaeal species (Figure 4). CYP174 and CYP1002 have three and four members on plasmids and 32 and 18 members on chromosomes (Figure 4). In archaea, these two P450 families have the highest number of members (Figure 2), suggesting members of these two families indeed expanded after transfer from plasmids to chromosomes.

**Figure 5:**
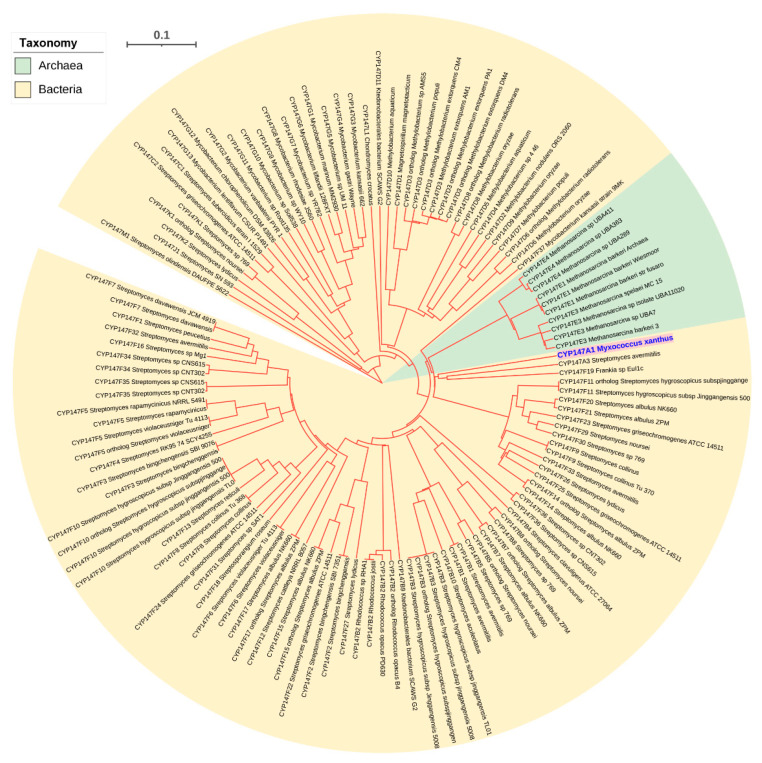
Phylogenetic analysis of the CYP147 P450 family members from archaea and bacteria. Different colors highlighted CYP147 members from *Methanosarcina* and CYP147A1 from *Myxococcus xanthus*. The tree branch length is indicated as the scale bar in the upper left corner. CYP147 members used in constructing the phylogenetic tree are presented in Table S7. The protein sequences were aligned by MAFFT v6.864 [36] and the tree was inferred by the Trex web server [37].

Among the P450 families shared between archaea and bacteria, CYP109 and CYP197 P450s are copresent on four different plasmids of four different archaeal species, indicating that these P450s might have come together as they are copresent in bacterial species as well (Figure 3 and Table S3). However, the third P450 family shared by these groups, CYP147, is not found on archaeal plasmids. In order to understand the origin of CYP147 in archaea, we analyzed the CYP147 family across living organisms (Figure 5 and Table S7). The analysis revealed that only nine bacterial genera (*Myxococcus*, *Streptomyces*, *Rhodococcus*, *Ktedonobacterales*, *Magnetospirillum*, *Methylobacterium*, *Mycobacterium*, *Frankia* and *Chondromyces*) have the CYP147 P450 family belonging to the subfamilies ranging from A–D and F–L (Table S7) [18]. Most of the CYP147 P450s belonging to the same subfamily were found to be orthologs indicating their origin from a common ancestor in these genera [18]. *Methanosarcina* of archaea has CYP147 subfamily E, which is not present in bacteria. All CYP147s of *Methanosarcina* belong to this same subfamily E and all share >85% sequence identity, strongly indicating their common ancestral origin. Interestingly, if one indel is removed, all CYP147E P450s of *Methanosarcina* are 60–61% identical to CYP147A1 of *Myxococcus xanthus*, suggesting they belong to the same subfamily. This was clear as the P450s of *Methanosarcina* and CYP147A1 of the *Myxococcus xanthus* group aligned next to each other on the phylogenetic tree (Figure 5). Based on these results, it seems probable that the CYP147E sequences of *Methanosarcina* originated in *Myxococcus* sp. Interestingly, the CYP147 gene was laterally transferred into *M. barkeri* after the acetate kinase (*ackA*) and phosphate acetyltransferase (*Pta*) genes were transferred about 250 million years ago as *M. mazei* and *M. acetivorans* have *ackA* and *Pta* genes [39] but not CYP147. Considering only a few CYP147 P450s exist in archaea, it is highly likely that this P450 also came via plasmids like CYP109 and CYP197 P450s.

### 2.4. Archaeal P450s Are Bacterial in Origin, Not Vice Versa

The polarity of lateral transfer (from bacteria to archaea) is partly based on the diversity of the P450s inside archaea (34 P450 families) and outside in bacteria (1910 P450 families) (Table 1). There are 130 CYP109s in archaea in 19 subfamilies. There are 183 CYP109s in bacteria in 41 subfamilies [18]. CYP147E is the only subfamily in archaea and is only in *Methanosarcina* (Figure 5). Bacteria have 11 subfamilies and CYP147E should belong to CYP147A in *Myxococcus* (Figure 5). CYP197 has 135 sequences in eight subfamilies in archaea, but most of the sequences are in CYP197C (87 sequences, probable orthologs) and CYP197L (35 sequences, probable orthologs) (Table S2). The other subfamilies have three members (CYP197AK) or only one sequence of each or just pseudogene fragments (Table S2). Contrary to what was observed in archaea, CYP197 in bacteria has 27 subfamilies [18].

### 2.5. Lateral Transfer of Putative Redox Partners Is Independent of P450s

**Figure 6:**
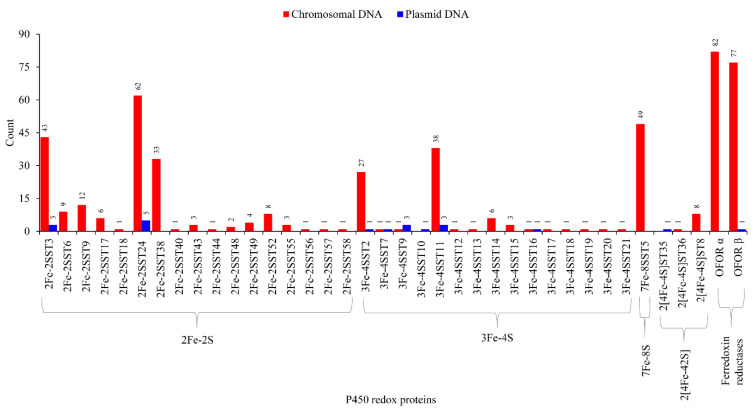
Comparative analysis of ferredoxins and ferredoxin reductase (OFOR) between chromosomal and plasmid DNA. Ferredoxin types and subtypes and OFOR alpha and beta subunits were presented in the figure. Detailed information is provided in Tables S5 and S6.

Genome-wide analysis revealed the presence of 352 ferredoxins belonging to four types such as 2Fe-2S, 3Fe-4S, 7Fe-4S and 2[4Fe-4S] in 40 archaeal species (Figure 6 and Tables S5 and S6). These 40 species were chosen because they have P450s on their plasmids (Table S4) and are thus appropriate for studying P450 and ferredoxin evolutionary links if any are present. Among ferredoxin types, 2Fe-2S had the highest number of members (199), followed by 3Fe-4S (94 members), 7Fe-4S (49 members) and 2[4Fe-4S] (Figure 6). Ferredoxin subtype analysis revealed archaeal species’ preference for specific subtypes (Figure 6), as observed in other microbes such as bacteria [34,40]. 2Fe-2S had 17 subtypes, where subtype 24 had the highest number of members (67) and 3Fe-4S had 15 subtypes, where subtype 11 had the highest number of members (41) (Figure 6). OFOR subunits can be found in archaeal genomes with 82 alpha and 78 beta subunits (Figure 6 and Tables S5 and S6). As indicated in the literature [31], our study found that these units’ corresponding genes were next to each other (Table S6). Almost all OFOR subunits were found on the chromosomal DNA, with only one exception of a single beta subunit on the *Haloprofundus salinisoli* strain SQT7-1 plasmid (NZ_CP083664.1) (Figure 6).

Ferredoxin subtype comparative analysis revealed plasmids and chromosomes share eight subtypes (Figure 6). Although archaea contained the same six ferredoxin types as bacteria, archaea had a low ferredoxin subtype diversity (Table S8). The ferredoxin subtype count between archaea vs. bacteria is as follows: 2Fe-2S 27 vs. 35; 3Fe-4S 8 vs. 16; 4Fe-4S 4 vs. 11; 7Fe-8S 4 vs. 6; 2[4Fe-4S] 21 vs. 21; 2[4Fe-4S]Alv 2 vs. 10 (Table S8).

The operonic analysis revealed that many P450s, both on the plasmids and chromosomes, are part of operons (Table 2). However, redox partners (ferredoxin or OFOR) are not typically part of these operons (Table 2). One interesting observation is the association between CYP174, 3Fe-4SST1 and 7Fe-8SST5 (Figure 3). In most archaeal genomes, CYP174 is located with 3Fe-4SST1 and 7Fe-8SST5 and, in some cases, one of these ferredoxins (Figure 3). In a few archaeal genomes, other P450s and redox partners were found between them (Figure 3).

### 2.6. CYP109, CYP147 and CYP197 Gave Rise to Archaeal P450s

From the results presented in this study, it is clear that only three P450s are common between bacteria and archaea, indicating a possibility that these three P450s gave rise to all P450s in archaea. However, we need phylogenetic evidence to conclude that these three P450 families led to the evolution of other archaeal P450 families. Thus, we have performed a phylogenetic analysis of archaeal P450s (Figure 1). The structure of the tree shows two main branches. CYP109 and CYP147 are on Branch A and CYP197 is on Branch B. Each branch could have diverged from a single donor (CYP197 in Branch B) or two (CYP109 and CYP147 in Branch A). BLAST analysis of Branch B P450s at the National Center for Biotechnology Information (NCBI) [41] gave top non-archaeal hits that are the best BLAST hits to multiple CYP197 sequences of bacteria (results not shown, see methods), suggesting P450s of Branch B are indeed descendants of CYP197 from bacteria.

One would anticipate that if a P450 is a progenitor for other P450s, it should appear deepest on the branch. These three P450 families, however, do not belong to the deepest branches (Figure 1). Initially, the deepest branches seem to be the donors. However, when you consider that gene duplication followed by the acquisition of new functions leads to divergence in the sequence, deeper branches can be evolved from branches later in the tree. One P450 example we can give is the CYP51 and CYP61/CYP710 families. These are both in the sterol biosynthesis pathway. CYP51 is a demethylase [9,42] and CYP61/710 is a desaturase that acts later in the pathway [43,44]. It is highly likely that CYP61/710 evolved from a gene duplication of the CYP51 as the sterol pathway was evolving [45,46]. Today these are in different families. Even CYP61 (fungi) and CYP710 of plants and some protists were initially placed in separate families, though they are now recognized as homologs.

### 2.7. Most of the Archaeal P450s Are Orphans with No Known Function

The catalytic activity of only one archaeal P450, CYP119, has been described. CYP119 is found to be catalytically diverse, including its peroxidase activity utilizing H_2_O_2_ [26]. CYP119 catalyzes the oxidation of lauric acid [47,48], epoxidation of styrene [26], chemical dehalogenation [49], electrochemical reduction of nitrite, nitric oxide and nitrous oxide [50] and peroxidation of Amplex^®^ Red [51]. CYP119 fused to proliferating cell nuclear antigen (PCNA) was shown to be more active in the hydroxylation of lauric acid due to the localization of ferredoxin and ferredoxin reductase via PCNA [52]. However, the natural substrates of CYP119 are not identified. Based on the characterized homologs in bacteria, CYP109 family members are involved in the oxidation of substrates such as n-alkanes, fatty acids, primary n-alcohols, terpenoids, testosterone and norisoprenoids [53,54,55]. CYP109B1 from *Bacillus subtilis* oxidizes saturated fatty acids along with their methyl and ethyl esters [54]. CYP109C2 and CYP109D1 of *Sorangium cellulosum* (delta proteobacteria) accomplish subterminal hydroxylation of saturated fatty acids [53]. CYP109D1 of *S. cellulosum* was also shown to have highly regioselective hydroxylation of norisoprenoids, alpha- and beta-ionone [56]. CYP109E1 from *Bacillus megaterium* catalyzes a cholesterol and vitamin D2 two-step hydroxylation at positions C24 and C25 [57,58].

CYP147G1 from *Mycobacterium marinum* has activity against fatty acids, specifically linear and ω-2 methyl branched fatty acids at the ω-1 position [59]. CYP147F1 from *Streptomyces peucetius* is an efficient long-chain fatty acid hydroxylase [60]. Many CYP147 and CYP197 members have been shown to be part of biosynthetic gene clusters in mycobacterial-, streptomyces- and firmicutes species, indicating their role in the biosynthesis of natural metabolites [61,62,63,64]. Apart from its involvement in natural metabolite biosynthesis, nothing is known about the functions of CYP197. The commonality seems to be fatty acids are preferred substrates.

## 3. Materials and Methods

### 3.1. Species and Their Genome Database Information

Archaeal genomes available for public use at the Joint Genome Institute Integrated Microbial Genomes and Microbiomes (JGI IMG/M) [35] were used in the study (last accessed on August 2022). The genome sequences include complete genomes, uncultured archaeal sequences and sequences from metagenomic studies. Information on the archaeal species used in the study is provided in Table S1.

### 3.2. Genome Data Mining and Annotation of P450s

Genome data mining and identification of P450s in archaea were carried out following the protocol described elsewhere [63,65]. Each archaeal sequence available at JGI IMG/M [35] was searched for P450s using the InterPro code “IPR001128”. The hit protein sequences were then searched for the presence of P450 characteristic motifs such as EXXR and CXG [66,67]. Proteins with no motifs and a short amino acid sequence length (<350 amino acids in length) were considered P450 fragments. These P450 fragments were not included in further analysis. Only five fragments were identified in the study. The rest of the P450s (1204) were selected for assigning the family and subfamilies. Following the International P450 Nomenclature Committee rule [14,15,17], proteins with >40% identity and >55% identity will be grouped under the same family and subfamily, respectively. P450s with less than 40% identity were assigned to a new P450 family. Archaeal P450s identified in this study and their protein sequences, assigned names and species are presented in Table S1.

### 3.3. Analysis of P450s in Archaeal Plasmids

Each archaeal plasmid’s (Table S4) proteome was manually searched for P450s. When a P450 was found, it was assigned to a family and subfamily, as described in the above section.

### 3.4. Phylogenetic Analysis of P450s

Phylogenetic analysis of P450s was carried out following the procedure described elsewhere [68]. The phylogenetic tree of P450s was constructed using protein sequences (Table S1). CYP147F37 (formerly CYP147E2) of *Mycobacterium kansasii* was included in the analysis as a positive control to check the alignment with the same P450 families in archaea. Firstly, the protein sequences were aligned by the MAFFT v6.864 [36] in the Trex web server with default parameters [37]. The alignments were then automatically subjected to interpret the best tree using the Trex web server [37]. Finally, the best-inferred tree was visualized, colored and generated by the Interactive Tree Of Life (iTOL) [69]. This method was used for constructing trees for archaeal P450s (Figure 1) and CYP147 P450s (Figure 5).

### 3.5. BLAST Analysis of Archaeal P450 Families for Affinity to CYP109 or CYP197

Family representatives for each archaeal P450 family were blast searched against the NCBI nr database to find the best hit. This best hit was blast-searched against all named prokaryotic P450s (https://drnelson.uthsc.edu/p450seqs-dbs/, accessed on 18 February 2023) to find the strongest family match. For example, the sequences in Figure 1 between CYP147 and CYP109 have four families. One sequence from each family was searched as described. The result was that the strongest family affinity to named P450s was always CYP109. A similar approach was used for the families in the Branch B.

### 3.6. Comparative Analysis of P450s

For comparative analysis, P450s from bacteria and eukarya were retrieved from the published article [18] and used in the study. The prokaryotic P450s can be downloaded at the website: https://drnelson.uthsc.edu/p450seqs-dbs/, accessed on 18 February 2023.

### 3.7. Genome Data Mining and Annotation of Ferredoxins

Genome data mining and annotation of ferredoxins in 40 archaeal species were carried out following the methods described elsewhere [34] with slight modifications. Each archaeal proteome, including proteins on plasmids, was manually searched for iron-sulfur cluster proteins. The selected proteins were then subjected to protein BLAST at the National Center for Biotechnology and Information (NCBI) [70] against the Protein Data Bank (PDB) database [71] and analyzed for the presence of characteristic motif of ferredoxins using the InterPro database [72] and NCBI Conserved Domains Database (CDD) [73]. Proteins that had a hit against ferredoxins in the PDB database and have ferredoxin motifs, as indicated by different databases, were selected for further annotation. Annotation of ferredoxins (assigning Fe-S cluster subtypes) was carried out based on the characteristic spacing patterns between cysteine amino acids of the Fe-S cluster-binding motif, as described elsewhere [34]. Ferredoxins belonging to the new subtypes were assigned a unique subtype number that corresponded to the continuation of ferredoxin subtype numbers published for *Bacteroidetes* species [40]. Because P450s are found in plasmids from 40 different species, ferredoxin analysis was restricted to these species to provide a clear picture of the ferredoxin origin and, if any, the relationship to P450s.

### 3.8. Comparative Analysis of Ferredoxins

For comparative analysis, ferredoxins from different domains of life were retrieved from the published articles [34,40] and used in the study. Using the ferredoxin subtype data generated in this study and from the published studies [34,40], a heatmap showing the presence and absence of ferredoxin subtypes in bacteria, archaea and eukarya was produced (Table S8).

### 3.9. Genome Data Mining of Ferredoxin Reductases

Genome data mining and annotation of ferredoxin reductases in 40 archaeal species were carried out with a manual search through the proteome. The proteins that are described as potential ferredoxin reductases in the literature [29] were selected and presented as putative ferredoxin reductases. As a potential ferredoxin reductase capable of transferring electrons to ferredoxins, we only found 2-oxoacid ferredoxin oxidoreductase (OFOR). Thus, in this article, we presented the alpha and beta subunits of OFOR.

### 3.10. Retrieving Protein Identification Numbers from NCBI

JGI IMG/M uses different protein identification numbers (IDs). Due to this reason, in this study, we retrieved protein IDs for P450s that are common between archaea and bacteria from NCBI. The GenBank accession numbers for the genomes, plasmids, P450s, ferredoxins and ferredoxin reductases (alpha and beta subunits) are listed in the supplemental tables.

### 3.11. Operon Predictions

Operons in archaeal genomes (plasmids and chromosomal DNA) were analyzed using Operon-mapper [74]. The complete gene sequence in FASTA format was downloaded (chromosomal and plasmid DNA) from NCBI and submitted to the Operon-mapper web server for operon prediction. The predicted operons were searched for the presence of P450s. The genes in the operons with P450s were noted and presented in the table format (Table 2).

## 4. Conclusions

Based on archaea having the lowest P450 diversity, saturation of P450 families and three common P450 families (CYP109, CYP147 and CYP197) between archaea and bacteria, we propose that archaea inherited P450s from bacteria by lateral gene transfer and it did not have any P450s originally. Only 34 P450 families are identified in archaea compared to over 1900 P450 families in bacteria (Table 1). One interpretation of this skewed abundance is that P450s came late to archaea, so they have not had as much time to diverge. This shows that archaeal P450s are of bacterial origin, not vice versa. The co-occurrence of CYP109 and CYP197 on the same plasmids raises the potential that both families might have been transferred to archaea at the same time. The presence and absence of CYP147 in *Methanosarcina* species may provide a date of transfer after the end Permian extinction event 250 million years ago. Phylogenetic analysis and the high affinity of diverged P450 families for one of the three families shared with bacteria indicates that CYP109, CYP147 and CYP197 gave rise to all archaeal P450s. Given the presence of ferredoxins of the same subtype on archaeal plasmids and chromosomes, as well as the same ferredoxin in bacteria and the low ferredoxin subtype diversity in archaea compared to bacteria, it is highly likely that ferredoxins were transferred laterally from bacteria to archaea, most likely via plasmids. However, annotating ferredoxin subtypes in all archaeal and bacterial species will reveal a clear picture of the genesis of ferredoxins in archaea. P450 redox partners (ferredoxins or OFOR) were not found to be part of P450 operons, indicating independent evolution of P450s and redox partners in archaea.

**Figure 7:**
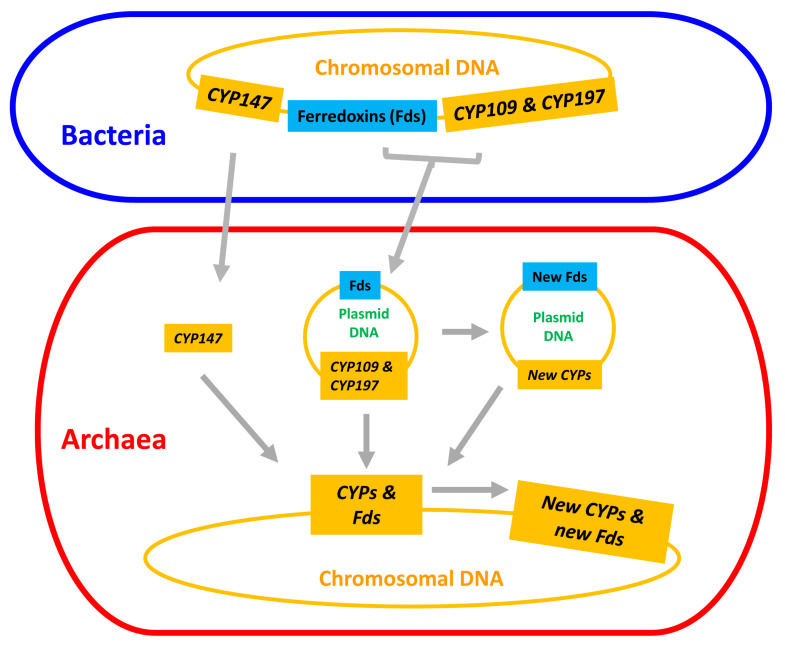
Schematic representation of evolution and diversification of P450s/CYPs and ferredoxins in archaea.

The emergence and divergence of P450s and ferredoxins in archaea can result in a number of scenarios: (i) plasmid-mediated direct transfer of these three P450 families (at least CYP109 and CYP197) and ferredoxins; (ii) the transferred P450s and ferredoxins gave rise to new P450s and new ferredoxins, either on plasmids or after transfer to chromosomal DNA; and (iii) subsequent divergence of new P450s and new ferredoxins resulted in the formation of all P450s and ferredoxins in archaea (Figure 7). A point to be noted is that all these scenarios are presented based on the available data as at the time of this publication. Thus, we do not rule out other lateral gene transfer mechanisms, but such mechanisms need evidence.

## Figures and Tables

**Table 1:** Comparative analysis of P450s and their families in species across biological kingdoms.

Species Category	Species Subcategory	Species Subcategory P450 Count	Species Category P450 Count	P450 Families
Animals			37,149	1948 *
	Mammals	4558		18
	Other vertebrates	3268		19
	Insects	22,173		1031
	Non-insect invertebrates	7150		880
Plants			42,102	819
Fungi			28,260	3204
Protozoa			5807	1374
Bacteria			17,236	1910
Archaea			1204	34
Viruses			37	13
Total			131,795	9302

Note: The data provided in the table for all categories except for archaea are from the previous report [18]. However, the information has been updated since 2018 with the latest numbers from the P450 library as of 28 November 2022 [16]. The symbol * indicates that families found in multiple groups are counted once, i.e., the total of families in all animals is less than the sum of the numbers for the individual groups of animals listed.

**Table 2:** P450 operon analysis in 40 archaeal species. Arrows indicate gene orientation.

Species Names	Accession Number	Operon
**Plasmid DNA**		
*Halorussus* sp. RC-68	NZ_CP035120.1	Epimerase/dehydratase→Thiamine pyrophosphate-binding protein→CYP109G24
*Halorussus* sp. YCN54	NZ_CP099994.1	Cupin domain-containing→ProteinNAD(P)-dependent oxidoreductase→CYP109G32
*Halorussus* sp. XZYJT49	NZ_CP096661.1	CYP174A39→PIN domain-containing protein→AbrB/MazE/SpoVT family DNA-binding domain-containing protein
*Haloprofundus salinisoli* strain SQT7-1	NZ_CP083664.1	Enoyl-CoA hydratase-related protein→CYP109G25
*Haloprofundus* sp. MHR1	NZ_MN918443.1	CYP109G22→Enoyl-CoA hydratase-related protein
*Haloprofundus halobius* strain SEDH52	NZ_CP083667.1	MFS transporter→CYP109G26
*Saliphagus* sp. WLHS1	NZ_CP100356.1	CYP1002B4→Putative protein
*Natrinema* sp. DC36	NZ_CP084474.1	CYP1014C9→Putative protein
*Halorubrum lacusprofundi* strain HLS1	NZ_KX906370.1	CYP109G27→SDR family oxidoreductase→Putative protein
*Halocatena* sp. AD-1	NZ_CP096021.1	ABC transporter permease subunit→CYP1014B9
	NZ_CP096022.1	Hydroxypyruvate isomerase→dehydrogenase→CYP109AU2
*Halobellus limi* CGMCC 1.10331	NZ_CP031313.1	Amidohydrolase family protein→CYP1002B4
**Chromosomal DNA**		
*Halorussus halophilus* strain ZS-3	NZ_CP044523.1	SAM-dependent methyltransferases→CYP1002C17
*Halorussus* sp. YCN54	NZ_CP099993.1	CYP1002C24→SAM-dependent methyltransferases
*Halorussus* sp. XZYJ18	NZ_CP100400.1	SAM-dependent methyltransferases→CYP1002C25
*Halorussus* sp. XZYJT49	NZ_CP096659.1	Dehydrogenase→Flavoprotein→CYP174C3
*Haladaptatus* sp. PSR5	NZ_CP085335.1	CYP174D1→Putative protein
*Halomicroarcula* sp. DT1	NZ_CP100404.1	Archaeal kinase→Mevalonate kinase→CYP174A38
*Halomicroarcula* sp. YSSS71	NZ_CP100407.1	Archaeal kinase→Mevalonate kinase→CYP174A38
*Halomicrobium salinisoli* strain TH30	NZ_CP084466.1	Putative protein→Putative protein→CYP174A35→Mevalonate kinase→Archaeal kinase
*Halomicrobium salinisoli* strain LT50	NZ_CP084463.1	Archaeal kinase→Mevalonate kinase→CYP174A36→Putative protein→Putative protein
*Halosiccatus urmianus* strain IBRC-M: 10911	NZ_CP084090.1	Archaeal kinase→Mevalonate kinase→CYP174A37→Putative protein→Tellurite resistance protein and related permeases→Ribosomal protein S2→Enolase→DNA-directed RNA polymerase, subunit K/omega→DNA-directed RNA polymerase, subunit N (RpoN/RPB10)→Ribosomal protein S9→Ribosomal protein L13→Ribosomal protein L18E→Putative protein
*Halalkalicoccus jeotgali* B3	NC_014297.1	Ketopantoate reductase→Zn finger protein HypA/HybF→CYP109G2
*Halocatena* sp. AD-1	NZ_CP096019.1	CYP109F16→Putative protein
*Halobellus limi* CGMCC 1.10331	NZ_CP031311.1	CYP1014A7→Phosphoribosylaminoimidazole (AIR) synthetase
*Halococcus dombrowskii* H4	NZ_CP095005.1	ATP-dependent 26S proteasome regulatory subunit→CYP109G4
*Halosegnis* sp. ZY10	NZ_CP101161.1	CYP2738A7→Putative protein
*Natribaculum breve* TRM20010	NZ_CP095390.1	Phytoene/squalene synthetase→CYP197C46
*Natronobiforma* sp. CGA73	NZ_CP101458.1	Predicted metal-dependent membrane protease→Holliday junction resolvase-archaeal type→Putative protein→CYP174B67
*Natronomonas* sp. ZY43	NZ_CP101154.1	CYP1603A5→Fibrillarin-like rRNA methylase→Protein implicated in ribosomal biogenesis, Nop56p
		Putative protein→Putative protein→CYP1002C26
		CYP1014G24→Aspartate/tyrosine/aromatic aminotransferase

Note: The chromosomal DNA of *Haloprofundus halobius* strain SEDH52 (NZ CP083666.1) has CYP-fragment1 as part of an operon with six other genes. This operon is not shown since this P450 is a fragment and may not be functioning. The protein names were the same as presented in the NCBI genome database and putative protein indicates that the protein’s function is unknown.

## Data Availability

Not applicable.

## Supplementary Materials

The following are available online at https://www.mdpi.com/article/10.3390/ijms24044161/s1.
