# Supplementary figures and images for: Evolution of Cytochrome P450 Enzymes and Their Redox Partners in Archaea
Source: Int J Mol Sci. 2023 Feb 19;24(4):4161. doi: 10.3390/ijms24044161 (PMC9962201; doi:10.3390/ijms24044161)

Figure S1

Number of sequences

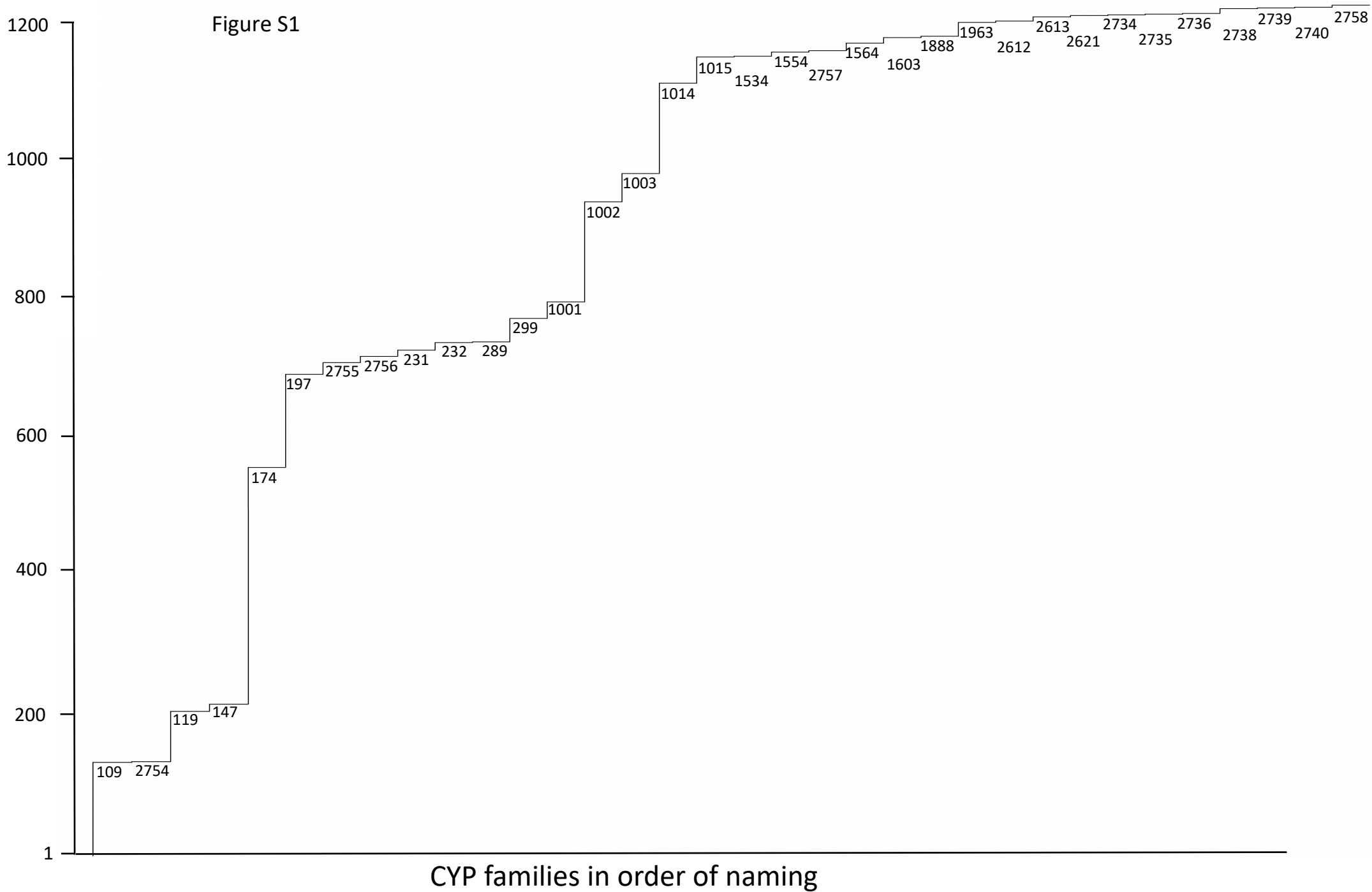

Supplement: Supplementary file 1 [file ijms-24-04161-s001.zip › Supplementary Information/Figure S1.pdf]
